# Nitric Oxide and Phytohormone Crosstalk Mediates Soybean Growth and Defence Against *Aphis glycines*

**DOI:** 10.1111/ppl.71070

**Published:** 2026-08-25

**Authors:** Fahad Ullah Khan, Muhammad Salman, Eun‐Woo Yu, Adil Hussain, Murtaza Khan, Byung‐Wook Yun

**Affiliations:** ^1^ Department of Applied Biosciences, College of Agriculture and Life Sciences Kyungpook National University Daegu South Korea; ^2^ Department of Plant Protection The University of Agriculture Peshawar Khyber Pakhtunkhwa Pakistan; ^3^ Department of Entomology Abdul Wali Khan University Mardan Mardan Khyber Pakhtunkhwa Pakistan; ^4^ Agriculture and Life Sciences Research Institute Kangwon National University Chuncheon Republic of Korea

**Keywords:** aphid resistance, defence‐related gene expression, nitric oxide, salicylic acid and jasmonic acid crosstalk, soybean

## Abstract

Aphid infestation, particularly by *Aphis glycines*, poses a major threat to soybean ( *Glycine max* ) production, necessitating sustainable defence strategies. This study evaluated the role of nitric oxide (NO) donor S‐nitrosocysteine (CySNO) in enhancing soybean resistance to aphids through hormonal crosstalk. A dose optimisation assay revealed that 0.1 mM CySNO significantly improved seed germination, shoot and root growth, while higher concentrations inhibited development. Greenhouse trials were then conducted by applying 0.1 mM CySNO directly to the roots, followed by foliar applications of salicylic acid (SA), jasmonic acid (JA) and their combination. Physiological parameters showed that CySNO pre‐treatment enhanced shoot length and leaf area, particularly under SA + JA application. Although chlorophyll content did not differ significantly, a slight positive trend was observed in all treated plants. Aphid population dynamics monitored over 20 days revealed that CySNO combined with SA + JA significantly suppressed aphid colonisation compared to individual treatments or controls. Quantitative real‐time PCR analysis demonstrated that CySNO upregulated key defence genes, including *GmGSNOR1*, *GmNOX1*, *GmPR1*, *GmLOX2* and *GmLOX10*, indicating enhanced activation of SA‐ and JA‐mediated pathways. Together, these results demonstrate that NO enhances soybean growth and strengthens defence responses via hormonal signalling, highlighting its potential as an effective strategy for aphid management within integrated pest management frameworks.

## 1 Introduction

Soybean aphid (*Aphis glycines* Matsumura) is a major pest of soybeans ( *Glycine max* [L.] Merrill), posing a serious threat to crop productivity. Native to East Asia, it was first detected in North America in 2000, where it has since caused significant economic losses (Posadas et al. 2025). Feeding damage caused by *A. glycines* results in reduced photosynthesis, stunted growth, fewer pods and diminished seed weight. In addition, the accumulation of honeydew promotes sooty mould, further impairing plant function (Ferdous 2022). Unlike antibiosis and antixenosis, tolerance exerts minimal selection pressure on insect populations and is thus considered a more durable form of resistance (Natukunda and MacIntosh 2020). Herbivore attacks, including aphid feeding, activate a cascade of local and systemic responses in plants, often mediated by hormonal signalling networks involving salicylic acid (SA), jasmonic acid (JA), ethylene (ET) and abscisic acid (ABA) (Yao et al. 2020). These phytohormones coordinate downstream defences such as cell fortification and the synthesis of secondary metabolites. Notably, aphid feeding elicits both SA‐ and JA‐mediated responses. The specific processes and outcomes, however, depend on factors such as the aphid species, plant genotype and whether the interaction is compatible or incompatible (Ali et al. 2024).

Nitric oxide (NO) is an important component of early defence signalling during aphid infestation, contributing to the oxidative burst, calcium mobilisation and transcriptional activation of defence‐related genes. Its interaction with hormonal pathways, particularly SA and JA, can either synergise or antagonise plant defence responses depending on the context (Khan et al. 2023). Literature suggests that NO modulates stomatal closure, hypersensitive response (HR)‐like cell death and PR protein expression mechanisms that can potentially influence soybean resistance to aphids (Wang et al. 2013).

Although considerable progress has been made in developing aphid‐resistant cultivars and integrated pest management strategies, there remains a need for deeper insight into the molecular crosstalk between NO and hormone‐mediated pathways during aphid attack. Understanding how NO integrates with phytohormone signalling to regulate soybean defences will not only advance our knowledge of plant–insect interactions but will also inform breeding strategies aimed at reducing pesticide reliance and enhancing sustainable resistance. Therefore, this study aimed to elucidate the role of NO‐mediated signalling in regulating hormone‐dependent defence responses during soybean aphid infestation. Using the NO donor S‐nitrosocysteine (CySNO), applied directly to the root zone as a root drench, we investigated its effects on SA‐ and JA‐associated defence responses and soybean resistance to aphids. To address this objective, we assessed aphid dynamics, plant growth performance, chlorophyll content and the transcriptional responses of key defence‐ and NO‐related genes, including *LOX2, LOX10, PR1, GmNOX1* and *GmGSNOR1*.

## 2 Materials and Methods

### 2.1 CySNO Dose Optimisation and Screening Assay

A screening experiment was conducted to determine the optimal concentration of CySNO to enhance seed germination and early seedling growth in soybean ( *Glycine max* L., cv. Daechan). Soybean seeds were surface sterilised using 2.5% sodium hypochlorite (NaOCl) for 20 min on a shaker (120 rpm), followed by three rinses with autoclaved distilled water. The surface‐sterilised seeds were then divided into six treatment groups: unprimed control, Hydro‐primed (soaked in distilled water), 10 mM 2‐(4‐Carboxyphenyl)‐4,4,5,5‐tetramethylimidazoline‐1‐oxyl‐3‐oxide (cPTIO), a NO scavenger and CySNO at concentrations of 0.1, 0.25 and 0.5 mM. Seeds were primed in the respective solutions for 12 h in the dark at room temperature and subsequently incubated on moist filter paper under controlled conditions (25°C ± 2°C, 16/8 h light/dark photoperiod). Based on the results of this screening assay, 0.1 mM CySNO was identified as the optimal concentration. For the subsequent aphid infestation experiment, a separate batch of soybean seeds was grown under greenhouse conditions, and 0.1 mM CySNO was applied as a root drench to the root zone.

### 2.2 Parameters Measured for Screening Assay

Germination was monitored daily for up to 5 days, and the germination percentage was determined by radicle emergence. After 7 days, shoot length (cm) was measured from the base of the hypocotyl to the tip of the first fully expanded true leaf, whereas root length (cm) was measured from the hypocotyl–root junction to the tip of the primary root using a ruler (Domingues Neto et al. 2024).

### 2.3 Plant Growth and Experimental Conditions

Soybean seeds (cv. Daechan) were obtained from the Common Genetic Resource Facility at Kyungpook National University, Republic of Korea. After surface sterilisation using 2.5% NaOCl for 20 min on a shaker (120 rpm), followed by three rinses with autoclaved distilled water, seeds were sown in plastic nursery pots (100 mm diameter × 90 mm height; approximately 550 mL volume). Each pot was filled with approximately 500 g of sandy soil. The seeds were germinated in plastic pots containing nutrient‐rich soil [peat moss (10%–15%), perlite (35%–40%), coco peat (45%–50%), zeolite (6%–8%), NH_4_^+^ (0.09 mg g^−1^), NO_3_ (0.205 mg g^−1^), P_2_O_5_ (0.351 mg g^−1^) and K_2_O (0.1 mg g^−1^)] in greenhouse with a (25°C ± 2°C, 14 h light/10 h dark photoperiod) temperature and 60%–70% relative humidity and Proper irrigation was performed whenever necessary. Typically, plants were watered every morning with 20 mL of tap water per pot. The CySNO concentration used in this experiment (0.1 mM) was selected based on the dose optimisation and screening assay described in Section 2.1. For the aphid infestation experiment, 0.1 mM CySNO was applied as a root drench to the root zone. Each plant received approximately 10 mL of CySNO solution once daily in the morning for five consecutive days. This application time was selected to minimise photodegradation and enhance absorption. Plants were then maintained under greenhouse conditions for 24 h before SA and JA treatments were applied.

### 2.4 Hormone Treatments Following CySNO Pre‐Treatment

To evaluate the effect of CySNO on hormone‐mediated defence responses, soybean plants at the V3 vegetative growth stage (three fully developed trifoliate leaves) were used. Based on the results of the dose optimisation assay, CySNO was applied directly to the root zone at a concentration of 0.1 mM (10 mL per plant per day) for five consecutive days. Twenty‐four hours after the final CySNO application, plants were treated with exogenous salicylic acid (SA; ≥ 99% purity, Sigma‐Aldrich) and jasmonic acid (JA; ≥ 95% purity, Sigma‐Aldrich). Stock solutions of SA and JA were prepared by dissolving each compound in a small volume of ethanol, followed by dilution with sterile distilled water containing 0.02% (v/v) Tween‐20 to obtain the desired working concentrations. For preparation of the working solutions, 13.8 mg SA was dissolved in sterile distilled water and adjusted to a final volume of 100 mL, whereas 2.1 mg JA was dissolved in sterile distilled water and adjusted to a final volume of 100 mL. Tween‐20 was added at a final concentration of 0.02% (v/v), corresponding to 20 μL per 100 mL solution. Fresh solutions were prepared immediately before application. Plants were assigned to eight treatment groups: (1) control (no CySNO, SA, or JA treatment), (2) SA, (3) JA, (4) SA + JA, (5) CySNO, (6) CySNO + SA, (7) CySNO + JA and (8) CySNO + SA + JA (Nyzhnyk et al. 2025). SA and JA were applied as foliar sprays using a fine‐mist sprayer (10 mL per plant) once daily for 5 consecutive days. In combination treatments, SA and JA were applied sequentially. Plants were maintained under greenhouse conditions throughout the experimental period.

### 2.5 Aphid Infestation

Soybean plants (cv. Daechan) were infested with *Aphis glycines* obtained from laboratory‐maintained colonies originally established from field collections. Two main experimental groups were established: plants without CySNO treatment and plants receiving CySNO treatment. Based on the results of the CySNO dose optimisation assay, CySNO was supplied in the root zone at 0.1 mM (10 mL per plant per day) for five consecutive days. Twenty‐four hours after the final CySNO application, plants were treated with SA, JA, or SA + JA as described above. Each treatment consisted of eight biological replicates arranged in a completely randomised design. Following completion of the hormone treatment, 15 adult aphids were manually introduced onto each plant (five aphids per trifoliate) according to standardised infestation procedures. Aphid populations and plant responses were subsequently monitored for 5 days after infestation (Nyzhnyk et al. 2025).

### 2.6 Data Collection and Growth Parameters

Five days after the final hormone treatment, plant growth parameters were recorded. Shoot length was measured using a ruler from the base of the stem at the soil surface to the tip of the youngest fully expanded trifoliate leaf and recorded in centimetres (cm).

### 2.7 Aphid Population Dynamics

Aphid counts were conducted on days 5, 10, 15 and 20 post‐infestations. Adult aphids visible on leaf surfaces were counted using a hand lens, whereas nymphs and aphids located in densely colonised areas were counted under a stereomicroscope to improve counting accuracy. Both adults and nymphs were counted across all trifoliate leaves, and the total number of aphids per plant was recorded according to established protocols (Beckendorf et al. 2008).

### 2.8 Chlorophyll Content Measurement

Chlorophyll content was measured using a chlorophyll concentration metre (MC‐100, Apogee Instruments Inc.). Measurements were taken from randomly selected leaves in triplicate, and the results were expressed as the Chlorophyll Concentration Index (CCI), a reliable proxy for physiological performance under stress conditions (Masaló and Oca 2020).

### 2.9 Quantitative Real‐Time PCR (qRT‐PCR) Analysis

To assess the expression of defence‐related genes, total RNA was extracted from leaves collected from all treatment groups (with and without CySNO pre‐treatment) using TRIzol reagent. Approximately 100 mg of leaf tissue was ground in liquid nitrogen and homogenised in 1 mL of TRIzol solution, followed by chloroform extraction, isopropanol precipitation and ethanol washing. RNA pellets were resuspended in 50 μL of nuclease‐free water and quantified. First‐strand cDNA was synthesised from 2 μg of total RNA using the BioFact RT Kit (BioFact, Korea). Quantitative real‐time PCR was conducted using an Eco Real‐Time PCR System (Illumina, USA) with BioFact 2X Real‐Time PCR Master Mix. Reactions were performed with 100 ng of cDNA and 10 nM of each primer. Gene‐specific primers (Table S1) were used to amplify *LOX10*, *LOX2*, *PR1*, *NOX1*, GS*NOR1* and the reference gene *ELF1*.

### 2.10 Statistical Analysis

Preliminary experiments conducted to determine the optimal CySNO concentration were performed with three independent replicates. The subsequent aphid infestation experiment was conducted with eight biological replicates per treatment group. Statistical analysis was performed using two‐way analysis of variance (ANOVA), followed by Bonferroni's post hoc test for multiple comparisons. Differences were considered statistically significant at *p* < 0.05 (*), *p* < 0.01 (**) and *p* < 0.001 (***). All statistical analyses and graphs were generated using GraphPad Prism version 9.0 (GraphPad Software).

## 3 Results

### 3.1 CySNO Screening

**FIGURE 1:**
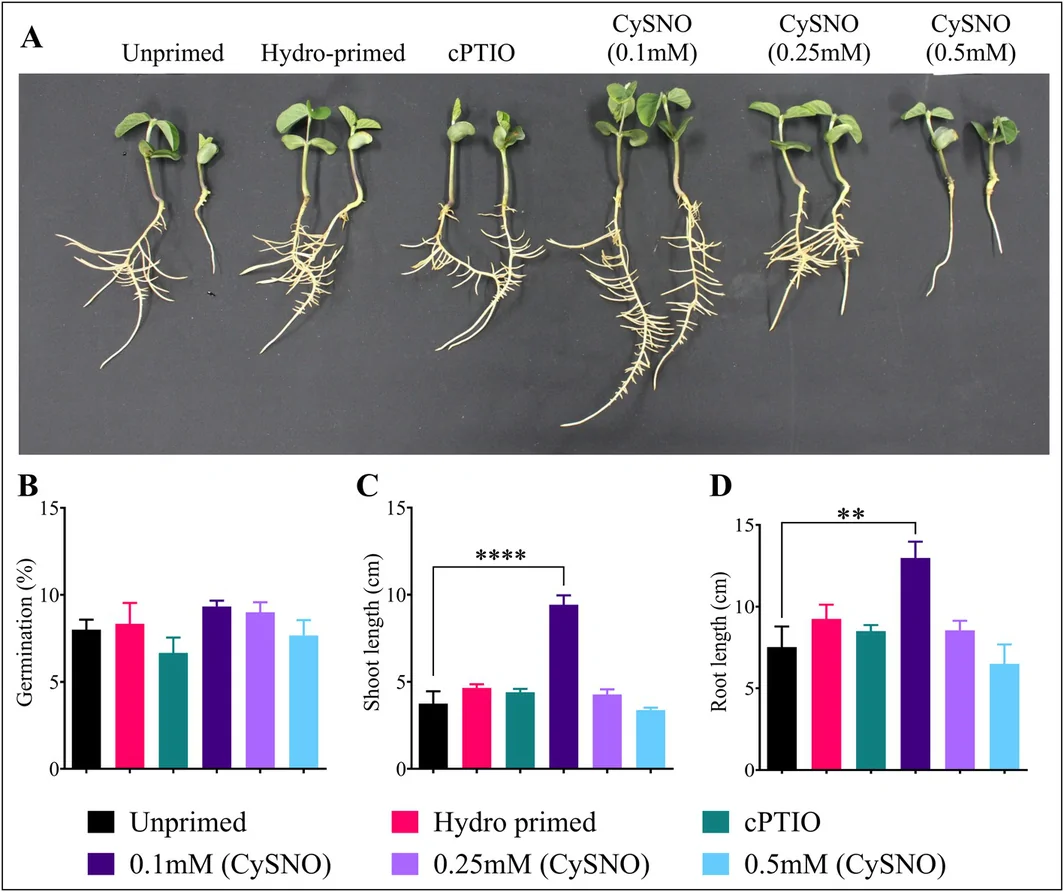
Effect of CySNO priming on soybean seedling development. Germination percentage (B), shoot length (C) and root length (D) of soybean seedlings treated with various concentrations of CySNO. A significant increase in shoot and root lengths was observed at 0.1 mM CySNO compared with the control treatments. Each data point represents the mean of at least three biological replicates (*n* ≥ 3). Error bars indicate standard deviation. Asterisks denote statistically significant differences (*p < 0.05; **p < 0.01, ***p < 0.001) determined by two‐way ANOVA followed by Bonferroni's multiple comparison test.

Visual observations (Figure 1A) support the quantitative data, clearly illustrating enhanced growth in seedlings treated with 0.1 mM CySNO, whereas those treated with 0.5 mM CySNO exhibited stunted shoot and root systems. To identify the optimal concentration of CySNO to promote early growth in soybeans, a priming experiment was conducted with the following treatments: unprimed control, hydro‐primed, cPTIO and CySNO at concentrations of 0.1, 0.25 and 0.5 mM. The effects were assessed based on seed germination %, shoot length, and root length (Figure 1A–D). As shown in Figure 1B, germination percentage was slightly higher in all primed seeds compared to the unprimed control, with 0.1 mM CySNO yielding the highest value. However, no significant differences were detected among treatments.

Shoot length varied markedly across the treatments (Figure 1C). The 0.1 mM CySNO treatment significantly increased shoot length relative to both unprimed and hydro‐primed controls (*p* < 0.001). In contrast, higher concentrations of CySNO (0.25 and 0.5 mM) led to a notable reduction in shoot length.

A similar pattern was observed for root development (Figure 1D). The 0.1 mM CySNO treatment significantly enhanced root length compared to other treatments (*p* < 0.01), including the cPTIO group, which showed suppressed root growth. In contrast, 0.25 and 0.5 mM CySNO treatments markedly reduced root length, with the 0.5 mM group showing the most severe inhibition.

### 3.2 Effect of CySNO Pre‐Treatment and Foliar Application of SA and JA on Shoot Length, Leaf Area and Chlorophyll Content

**FIGURE 2:**
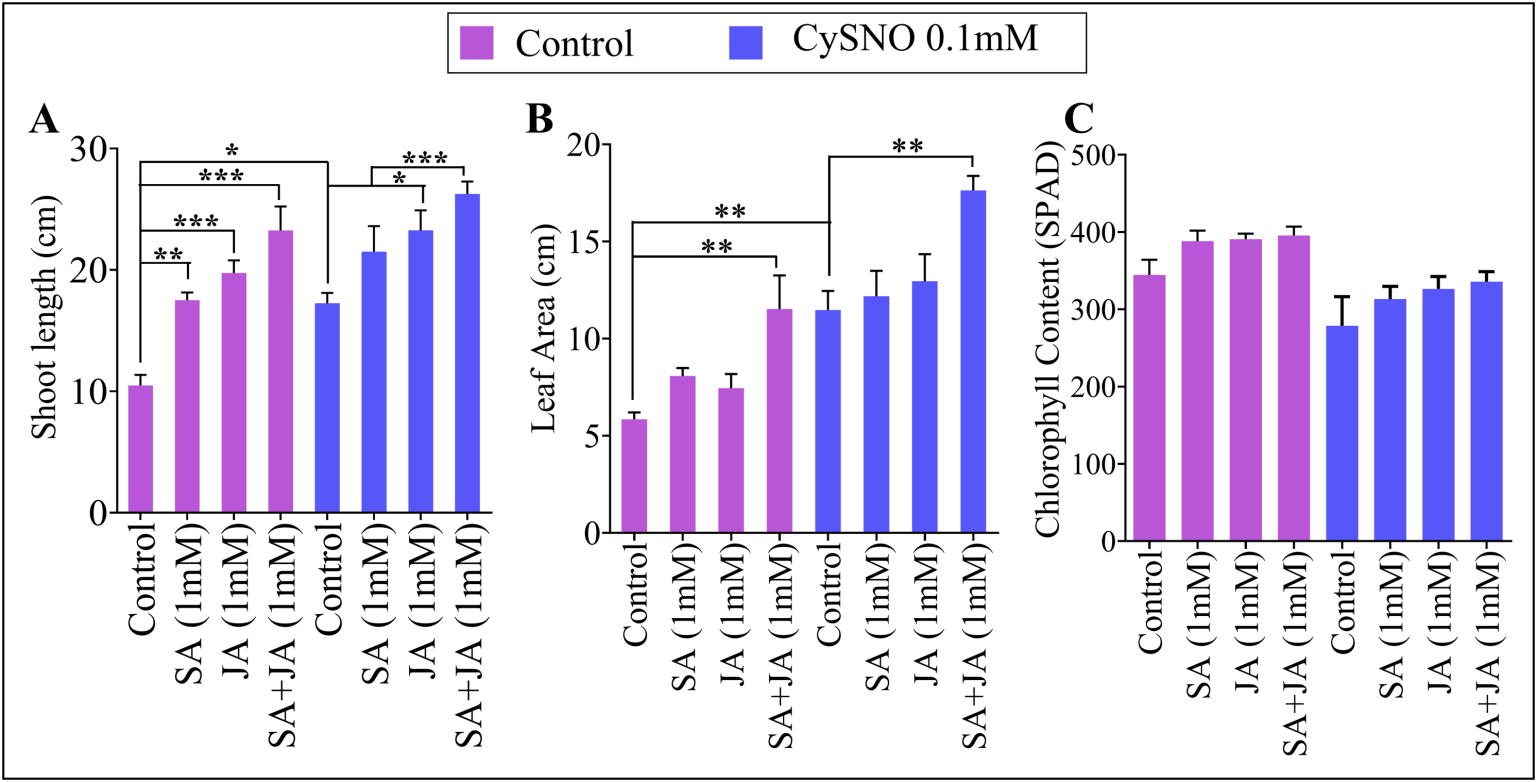
Effects of CySNO pre‐treatment and foliar application of Salicylic acid (SA), jasmonic acid (JA) and their combination (SA + JA). Shoot length (A), leaf area (B) and chlorophyll content (C) in soybean plants under soybean aphid infestation. Pre‐treatment with 0.1 mM CySNO, followed by applications of SA and JA (1 mM each), enhanced shoot growth and leaf area, with the most significant improvements observed in the combined SA + JA treatment group. While chlorophyll content showed no statistically significant differences, a slight upward trend was observed across all treated groups compared with the untreated control. Error bars indicate standard deviation. Asterisks denote statistically significant differences (*p < 0.05; **p < 0.01, ***p < 0.001) as determined by two‐way ANOVA followed by Bonferroni's multiple comparison test.

To evaluate the interactive effects of NO and phytohormonal treatments under aphid stress, soybean plants were pre‐treated with 0.1 mM CySNO and subsequently sprayed with 1 mM SA, 1 mM JA, or their combination (SA + JA). Growth parameters, including shoot length, leaf area and chlorophyll content, were measured to assess the physiological responses (Figure 2A–C).

As shown in Figure 2A, foliar application of SA or JA alone significantly enhanced shoot length compared to untreated controls (*p* < 0.01). However, CySNO pre‐treatment further amplified this effect across all groups. Among all combinations, the SA + JA treatment following CySNO pre‐treatment led to the most pronounced increase in shoot length (*p* < 0.01), indicating a strong interaction between NO signalling and hormone‐induced defence activation. Similarly, leaf area (Figure 2B) was significantly improved by SA + JA treatment even without CySNO pre‐treatment (*p* < 0.01). CySNO alone also increased leaf expansion, and the combined application of CySNO with SA + JA produced the highest leaf area among all treatments, thereby promoting vegetative growth.

In contrast, chlorophyll content (Figure 2C) did not differ significantly between treatments. However, a slight upward trend was observed in plants treated with SA, JA, or SA + JA, both with and without CySNO pre‐treatment, compared to the control. Although not statistically robust, this pattern suggests a mild enhancement of photosynthetic capacity under combined hormonal and NO treatment. Overall, these results indicate that 0.1 mM CySNO pre‐treatment enhances the effectiveness of SA and JA in promoting shoot and leaf development under biotic stress.

### 3.3 Effect of CySNO Pre‐Treatment and Hormonal Sprays on Soybean Aphid Population Dynamics

**FIGURE 3:**
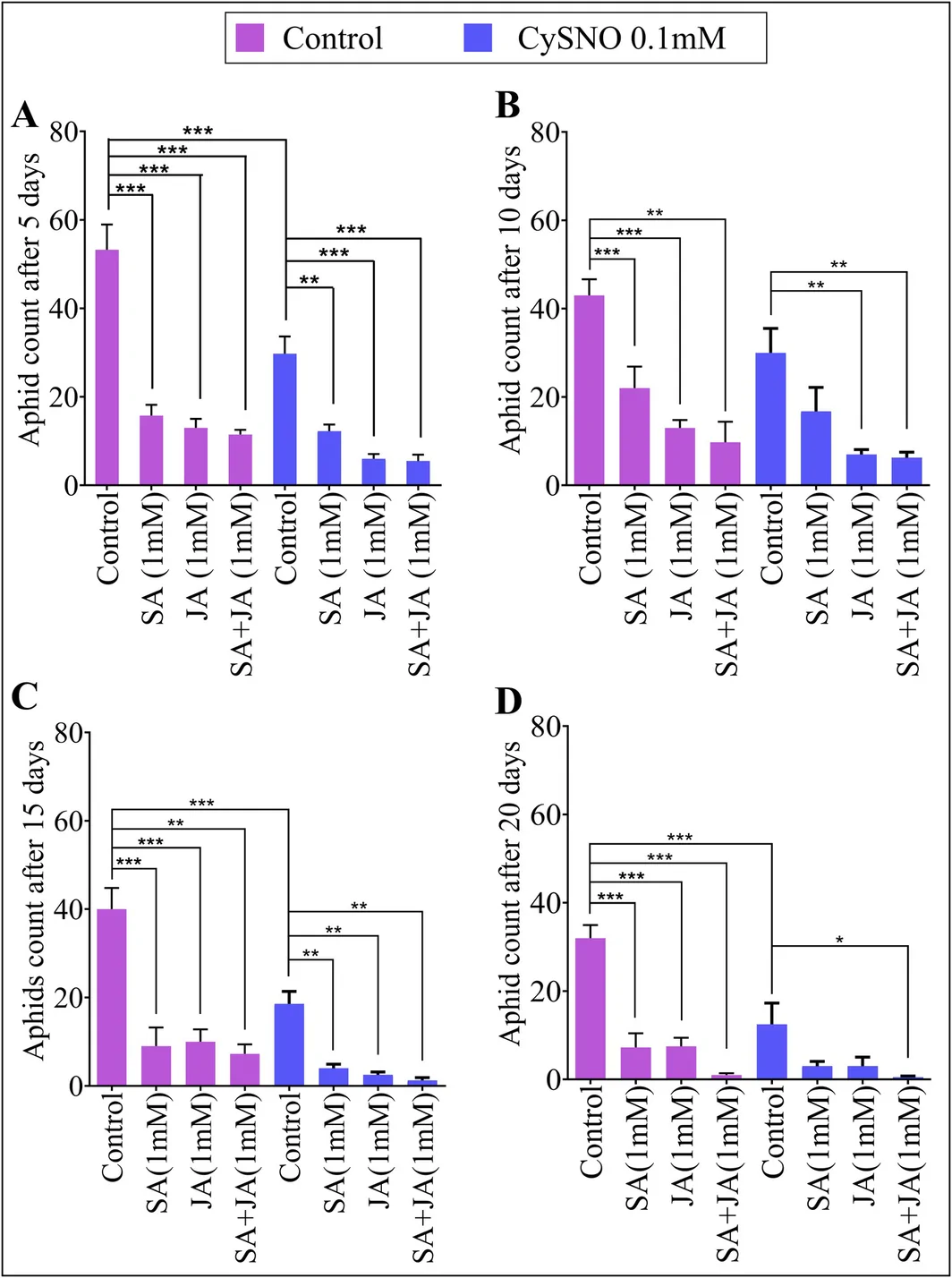
Effect of CySNO pre‐treatment and foliar application of Salicylic acid (SA) and jasmonic acid (JA) on the population dynamics of soybean aphids. Significant decreases in aphid populations were observed at 5 days (A), 10 days (B), 15 days (C) and 20 days (D) post‐infestation. Each data point represents the mean of at least three biological replicates (*n* ≥ 3). Error bars indicate standard deviation (SD). Asterisks (*) denote statistically significant differences (*p < 0.05; **p < 0.01, ***p < 0.001) as determined by two‐way ANOVA followed by the Bonferroni multiple comparison test.

To evaluate the efficacy of CySNO and hormonal treatments in suppressing soybean aphid populations, aphid numbers were monitored at 5‐day intervals up to 20 days post‐treatment (Figure 3A–D). Treatments included foliar application of SA, JA and their combination (SA + JA), with or without prior CySNO pre‐treatment. After 5 days (Figure 3A), all treatments—SA, JA and SA + JA—significantly reduced aphid populations compared to the untreated control (*p* < 0.001). CySNO pre‐treatment further enhanced the efficacy of each hormonal treatment. Notably, the greater reduction was observed in plants pre‐treated with CySNO and subsequently treated with SA + JA, indicating a strong interaction between NO signalling and phytohormonal defence pathways.

At 10 days post‐treatment (Figure 3B), aphid suppression remained more effective in CySNO‐pre‐treated plants. While SA and JA alone continued to significantly reduce aphid numbers compared to the control (*p* < 0.001), combining them with CySNO pre‐treatment resulted in an even greater reduction. Among these, CySNO + SA + JA again demonstrated the most pronounced suppression, although the difference from JA + CySNO was slightly less marked.

After 15 days (Figure 3B), the trend persisted: plants receiving CySNO pre‐treatment followed by hormonal application exhibited significantly lower aphid populations than non‐pretreated plants (*p* < 0.01, *p* < 0.001). The combined SA + JA treatment continued to show superior performance over single‐hormone applications. Importantly, even CySNO pre‐treatment alone conferred notable aphid suppression compared to untreated control.

By day 20 (Figure 3D), aphid populations remained lowest in plants treated with CySNO + SA + JA. Although reductions in CySNO + SA or CySNO + JA treatments were slightly less pronounced, they still significantly outperformed single‐hormone treatments without CySNO (*p* < 0.05). Control plants treated only with CySNO also showed moderate aphid suppression, confirming its independent protective effect. Together, these results clearly demonstrate that 0.1 mM CySNO pre‐treatment significantly enhances the effectiveness of SA and JA, particularly when applied in combination, in suppressing soybean aphid populations over time. The data support the interaction between NO and hormone‐induced defence mechanisms, leading to sustained pest suppression.

### 3.4 CySNO Pre‐Treatment Enhances Transcriptional Regulation of Defence Marker Genes Against Aphid Infestation in Soybeans

**FIGURE 4:**
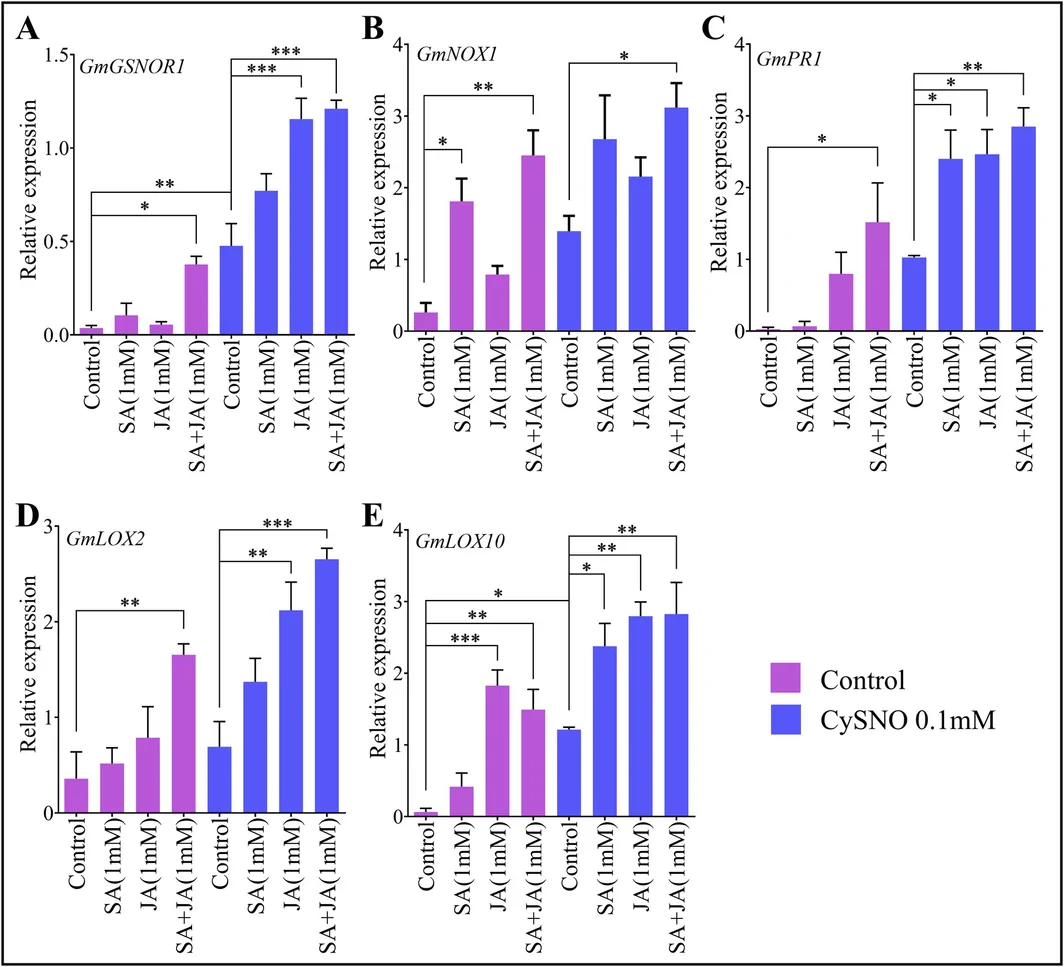
Pre‐treatment with nitric oxide followed by Salicylic acid and Jasmonic acid protects via transcriptional regulation of marker genes against aphid tolerance in soybeans. Pre‐treatment with CySNO followed by SA, JA, or their combination (SA + JA) resulted in a significant upregulation of nitric oxide‐related genes, including *GmGSNOR1* (A) and *GmNOX1* (B). Additionally, there was a marked increase in the expression of aphid‐responsive genes *PR1* (C), *LOX2* (D) and *LOX10* (E) in soybean. Each data point represents the mean of at least three biological replicates (*n* ≥ 3). Error bars indicate standard deviation (SD). Asterisks (*) denote statistically significant differences (*p < 0.05; **p < 0.01, ***p < 0.001) as determined by two‐way ANOVA followed by the Bonferroni multiple comparison test.

To elucidate the molecular basis of CySNO‐induced resistance against soybean aphids, we analysed the expression of key defence‐related genes: *GmGSNOR1*, *GmNox1*, *GmPR1*, *GmLOX2* and *GmLOX10* in response to foliar application of SA, JA and SA + JA, with or without CySNO pre‐treatment (Figure 4A–E). As shown in Figure 4A, CySNO pre‐treatment significantly upregulated *GmGSNOR1* expression compared to untreated controls (*p* < 0.01). Plants treated with JA or SA or JA + SA exhibited further enhancement in *GmGSNOR1* transcript levels when pre‐treated with CySNO (*p* < 0.001), compared to their respective treatments without CySNO. This suggests that NO signalling plays a central role in redox homeostasis and hormonal crosstalk during aphid stress.

Similarly, Figure 4B shows that *GmNox1* expression increased significantly following SA and SA + JA treatments. Notably, the expression was further elevated in plants treated with CySNO prior to SA + JA application (*p* < 0.05), reinforcing the involvement of NO in modulating SA‐mediated defence signalling. In Figure 4C, expression of the pathogenesis‐related gene *GmPR1* was moderately induced by SA and JA applications individually and further upregulated with CySNO pre‐treatment. The combined SA + JA treatment resulted in the highest expression levels, particularly in CySNO‐primed plants (*p* < 0.01), indicating adaptive effects on defence responses. The *GmLOX2* gene, a key component of the JA signalling pathway, was also significantly induced by the combined SA + JA treatment (Figure 4D). Expression was notably higher in plants pre‐treated with CySNO compared to those treated with hormones alone (*p* < 0.01), suggesting enhanced JA‐mediated defence via NO. Figure 4E demonstrates that *GmLOX10*, another JA‐responsive gene, was significantly upregulated by JA and SA + JA treatments. This upregulation was even more pronounced in CySNO‐pretreated plants across all hormonal treatments (*p* < 0.001), further supporting the role of NO in strengthening JA‐based defence pathways. Collectively, these results confirm that 0.1 mM CySNO pre‐treatment primes soybean defence mechanisms by enhancing the transcription of key marker genes involved in NO, SA and JA signalling. This transcriptional activation likely underlies the observed physiological resistance to aphid infestation.

## 4 Discussion

Aphids, particularly the soybean aphid (*A. glycines* Matsumura), are among the most destructive phloem‐feeding pests affecting soybean production globally. Their ability to cause significant yield losses, vector plant viruses and overcome host resistance has necessitated exploring more sustainable and systemic plant defence strategies (Ragsdale et al. 2007). In this context, our study highlights the role of NO delivered via CySNO as an effective agent that enhances early plant growth and activates robust defence responses against aphid infestation when used in conjunction with phytohormonal treatments. We began by identifying an optimal CySNO concentration (Figure 1A–D). Among the tested doses, 0.1 mM CySNO significantly increased shoot and root lengths (Figure 1A–D), whereas higher concentrations inhibited growth, likely due to nitrosative stress. These observations align with previous reports indicating that low concentrations of NO donors promote root architecture and seedling vigour by regulating auxin transport and cell expansion (Sanz et al. 2015), whereas excessive NO can generate reactive nitrogen species that impede growth (Fancy et al. 2017).

Building on this, we examined whether pre‐treatment with 0.1 mM CySNO could potentiate the effects of SA and JA under aphid stress. As shown in Figure 2, CySNO pre‐treatment significantly enhanced shoot length and leaf area across all hormone treatments, with the SA + JA combination yielding the most robust vegetative growth. The enhanced growth observed under combined treatments suggests a positive association between NO treatment and hormone‐related responses that contribute to improved leaf development (Shah et al. 2023). Interestingly, chlorophyll content did not vary significantly among treatments (Figure 2C), though a subtle trend of increased chlorophyll was observed with CySNO and hormone co‐treatment, suggesting limited but potential improvement in photosynthetic performance.

The physiological benefits conferred by CySNO were observed in its impact on aphid suppression (Figure 3A–D). Aphid population dynamics monitored over 20 days demonstrated that while SA, JA and their combination reduced aphid numbers on their own, their efficacy was significantly amplified when combined with CySNO pre‐treatment. The lowest aphid populations consistently occurred in the CySNO + SA + JA group, suggesting sustained defence activation. This effect may be attributed to NO‐mediated modulation of inducible defence pathways, resulting in a faster and stronger response upon pest attack (Arasimowicz and Floryszak‐Wieczorek 2007).

At the molecular level, we investigated the transcriptional regulation of key defence‐related genes (Figure 4A–E). CySNO pre‐treatment upregulated *GmGSNOR1*, a regulator of NO homeostasis, suggesting that exogenous NO induces a feedback mechanism to control cellular nitrosothiol levels and prevent toxicity. Likewise, *GmNox1*, a central node in SA‐mediated signalling, was more strongly induced when CySNO was combined with SA and JA, reinforcing NO's role in amplifying systemic acquired resistance (Feechan et al. 2005; Tada et al. 2008). The expression of *GmPR1*, *GmLOX2* and *GmLOX10* marker genes for SA‐ and JA‐mediated defence was also elevated under combined treatments, especially in CySNO‐pretreated plants, supporting a model in which NO coordinates both SA‐ and JA‐dependent defence responses to enhance resistance.

This dual activation is critical considering that SA and JA pathways often exhibit antagonistic interactions (Asgher et al. 2017; Spoel and Dong 2012; Vlot et al. 2009; Wasternack and Hause 2013). The direct impact of NO on phytohormone production and metabolism has been well investigated and reported in the literature. Through direct measurements, multiple papers from our lab have shown that NO regulates phytohormone production (Pande et al. 2022). NO also induces global transcriptomic changes involving genes responsible for NO production and metabolism (Hussain et al. 2016). The results of this study suggest that NO influences SA‐ and JA‐associated defence responses during aphid infestation, consistent with observations in Arabidopsis and tomato (Huang et al. 2004; Zhou et al. 2005). However, NO and phytohormone levels were not directly quantified in the present study; the precise mechanisms underlying NO–hormone crosstalk are still being unravelled and require further investigation (Chakraborty 2021; Mur et al. 2013). Additionally, the possibility of NO‐mediated modulation of volatile organic compound emissions or the recruitment of aphid natural enemies, though not evaluated here, suggests further experiments (Adams et al. 2015; Shapiro 2005). Collectively, this study demonstrates that CySNO pre‐treatment at 0.1 mM enhanced soybean early growth (Figure 1), promoted vegetative development under stress (Figure 2), suppressed aphid infestation (Figure 3) and increased the expression of defence‐related genes (Figure 4). These findings indicate that CySNO treatment was associated with improved plant performance and defence responses under aphid infestation. Further studies involving direct biochemical and molecular analyses are needed to elucidate the underlying mechanisms.

Considering these results, exogenous NO application to the root zone, combined with phytohormonal sprays, could be a promising tool in integrated pest management strategies to reduce chemical pesticide use. However, the practical application of such techniques will require validation across diverse environmental conditions, soybean cultivars and aphid biotypes. Future studies should also explore the long‐term persistence of NO‐induced resistance, its impact on beneficial insects and the underlying transcriptional and metabolic changes through omics‐based approaches.

## 5 Conclusion

This study demonstrates that 0.1 mM CySNO significantly enhances early seedling growth in soybean and enhances plant defence responses against aphid infestation. CySNO applied to the roots, especially when combined with foliar application of SA and JA, promotes shoot and root development, reduces aphid population growth and upregulates key defence‐related genes. The observed effects suggest that NO signalling interacts positively with phytohormonal pathways to strengthen both physiological and molecular resistance mechanisms. These findings support the potential application of NO‐based treatments as an environmentally friendly component of integrated pest management in soybean cultivation.

## Author Contributions

**Fahad Ullah Khan:** conceptualization, data curation, formal analysis, investigation, methodology, visualisation, writing – original draft. **Muhammad Salman:** methodology, validation, writing – review and editing. **Eun**‐**Woo Yu:** methodology, validation. **Adil Hussain:** methodology, validation, writing – review and editing. **Murtaza Khan:** validation, writing – review and editing. **Byung**‐**Wook Yun:** validation, resources, writing – review and editing, funding acquisition, project administration.

## Funding

This study was funded by the Basic Science Research Program of the Ministry of Education's National Research Foundation of Korea (NRF) under Grant No. RS‐2023‐00245922 to Byung‐Wook Yun and by the Korea Basic Science Institute (National Research Facilities and Equipment Center, 2021R1A6C101A416) funded by the Ministry of Education, biological materials specialized graduate program through the Korea Environmental Industry & Technology Institute (KEITI), funded by the Ministry of Climate, Energy and Environment (MCEE) and the ANCHOR Program through the Daegu ANCHOR Center, funded by the Ministry of Education (MOE) and the Daegu Metropolitan City, Republic of Korea (2026‐ANCHOR‐03‐001). This work was also supported by the KNU, Appointment and Support Program for Global JA Professors through the Regional Innovation System & Education (RISE) Global 30 Program through the Daegu RISE Center, funded by the Ministry of Education (MOE) and Daegu, Republic of Korea (2026‐Glocal30‐03‐001) awarded to Prof. Adil Hussain.

## Disclosure

The authors declare that no AI‐assisted technologies were used in the preparation of this manuscript.

## Conflicts of Interest

The authors declare no conflicts of interest.

## Supporting information


**File S1:** ppl71070‐sup‐0001‐Supplemental File.docx. **Table S1**. List of primers used for real‐time quantitative PCR.

## Data Availability

The data that supports the findings of this study are available in the Supporting Information of this article.
