# Supplementary material for: Nitric Oxide and Phytohormone Crosstalk Mediates Soybean Growth and Defence Against Aphis glycines
Source: Physiol Plant. 2026 Aug 25;178(5):e71070. doi: 10.1111/ppl.71070 (PMC13504698; doi:10.1111/ppl.71070)
Supplement: Supplementary file 1 [file PPL-178-e71070-s001.docx]

**Nitric oxide and phytohormone crosstalk mediates Soybean growth and defence against *Aphis glycines***

**Fahad Ullah Khan^1^, Muhammad Salman^2^, Eun-Woo Yu^1^, Adil Hussain^3*^, Murtaza Khan^4^ and Byung-Wook Yun^1*^**

**Table 1**. List of Primers used for real-time quantitative PCR.

| **Gene** | **Forward primer** | **Reverse primer** |
| --- | --- | --- |
| *GmGSNOR1* | GACCGGGATCACACCCATTT | ATTCCCACCACGTACCACAC |
| *GmNox1* | CAGAGCGCGGCTTTCACTTT | CACGTCATCGCTGTTGCTGT |
| *GmPR1* | GTGCAATGGAGTTTGTGGTC | TCACATAATTCCCACGAGGA |
| *GmLox2* | ATGGAAATCAACGCGCTTGC | TGCAGGTCGAATTGCCATTG |
| *GmLox 10* | TCTGCATCTCAAAATGTGATACCTC | CATCCATCCAGACAGATTCACTTG |
| *GmELF1* | CCACTGCTGAAGAAGATGATGATG | AAGGACAGAAGACTTGCCACTC |
